# FcγRIIB (CD32B) antibodies enhance immune responses through activating FcγRs

**DOI:** 10.1093/cei/uxaf015

**Published:** 2025-03-16

**Authors:** Alexander P Simpson, Robert J Oldham, Kerry L Cox, Martin C Taylor, Sonya James, Ann L White, Yury Bogdanov, Martin J Glennie, Björn Frendeus, Mark S Cragg, Ali Roghanian

**Affiliations:** Antibody and Vaccine Group, School of Cancer Sciences, Centre for Cancer Immunology, University of Southampton Faculty of Medicine, Southampton, Hampshire, UK; Antibody and Vaccine Group, School of Cancer Sciences, Centre for Cancer Immunology, University of Southampton Faculty of Medicine, Southampton, Hampshire, UK; Antibody and Vaccine Group, School of Cancer Sciences, Centre for Cancer Immunology, University of Southampton Faculty of Medicine, Southampton, Hampshire, UK; Antibody and Vaccine Group, School of Cancer Sciences, Centre for Cancer Immunology, University of Southampton Faculty of Medicine, Southampton, Hampshire, UK; Antibody and Vaccine Group, School of Cancer Sciences, Centre for Cancer Immunology, University of Southampton Faculty of Medicine, Southampton, Hampshire, UK; Antibody and Vaccine Group, School of Cancer Sciences, Centre for Cancer Immunology, University of Southampton Faculty of Medicine, Southampton, Hampshire, UK; Antibody and Vaccine Group, School of Cancer Sciences, Centre for Cancer Immunology, University of Southampton Faculty of Medicine, Southampton, Hampshire, UK; Antibody and Vaccine Group, School of Cancer Sciences, Centre for Cancer Immunology, University of Southampton Faculty of Medicine, Southampton, Hampshire, UK; Antibody and Vaccine Group, School of Cancer Sciences, Centre for Cancer Immunology, University of Southampton Faculty of Medicine, Southampton, Hampshire, UK; BioInvent International AB, Sölvegatan 41, Lund, Sweden; Antibody and Vaccine Group, School of Cancer Sciences, Centre for Cancer Immunology, University of Southampton Faculty of Medicine, Southampton, Hampshire, UK; Institute for Life Sciences, University of Southampton, Highfield, Southampton, UK; Antibody and Vaccine Group, School of Cancer Sciences, Centre for Cancer Immunology, University of Southampton Faculty of Medicine, Southampton, Hampshire, UK; Institute for Life Sciences, University of Southampton, Highfield, Southampton, UK

**Keywords:** Fc receptors, immunotherapy, antibodies, inhibitory/activating receptors, vaccination

## Abstract

Fc receptors (FcR) play a key role in coordinating responses from both the innate and adaptive immune system. The inhibitory Fc gamma receptor (FcγRIIB/CD32B; referred to as FcγRII/CD32 in mice) restrains the immune response, specifically through regulating immunoglobulin G (IgG) effector functions. FcγRII-deficient mice demonstrate elevated incidence and severity of autoimmunity and increased responses to immunization and infections. To explore the potential of FcγRIIB as a target for augmenting vaccines, we tested the ability of monoclonal antibodies (mAb) against mouse FcγRII and human FcγRIIB to enhance humoral responses in preclinical models. We used wild-type (WT), FcγR-deficient, and human FcγRIIB transgenic (Tg) mice with either a functional intracellular domain (hFcγRIIB Tg) or lacking immunoreceptor tyrosine-based inhibitory motif (ITIM) signalling capacity (NoTIM). Targeting mouse FcγRII and human FcγRIIB with antibodies significantly augmented humoral immune responses against experimental antigens and enhanced tumour clearance *in vivo*. Surprisingly, mAbs without a functional Fc (N297Q; referred to as Fc-null) lacked efficacy. Similarly, blocking FcγRII in mice lacking activating FcγRs failed to enhance immune responses. Conversely, blocking both signalling-competent and signalling-defective (NoTIM) FcγRIIB in Tg mice with a WT, but not Fc-null, FcγRIIB mAb equally enhanced immunity. These data indicate the redundancy of inhibitory signalling in potentiating immune responses *in vivo*. Collectively, our data suggest that mAb-targeting of FcγRIIB stabilizes mAb Fc and enhances immune responses via Fc-mediated crosslinking of activating FcγRs, irrespective of the inhibitory function of FcγRIIB. These findings support a strategy to boost immune responses in immunization protocols.

## Teaser

WT anti-mouse FcγRII and -human FcγRIIB mAbs augment humoral immune responses via interaction of their Fc with activating FcγRs. This study reveals that the enhancement of immune responses is not as a direct consequence of blocking FcγRIIB and its downstream ITIM-mediated signalling. Rather, their binding to FcγRIIB acts as an anchor to promote the engagement of adjacent (cis or trans) activating FcγRs via the mAb Fc domain, thereby inducing a more potent immune response upon immunization.

## Introduction

Fc gamma receptors (FcγR), expressed on various leukocyte subsets, are receptors for the Fc domain of IgG and can facilitate clearance of immune complexes (IC) and phagocytosis of opsonized targets (e.g. bacteria), among other important immunoregulatory functions [1–5]. There are six FcγRs in humans, four in mice, but only a single inhibitory receptor in each species; FcγRIIB (CD32B) [3, 6]. Both human FcγRIIB and mouse FcγRII are glycoproteins consisting of two Ig-like extracellular domains, a transmembrane region and a cytoplasmic tail containing an ITIM domain [2, 7]. In humans and mice, alternative splicing of FcγRIIB transcripts results in FcγRIIB1 and FcγRIIB2 isoforms. FcγRIIB1 has a longer cytoplasmic tail (19 and 47 amino acids in humans and mice, respectively), which impedes it from entering clarthrin-coated pits and prevents endocytosis/internalization of the receptor following engagement of IgG ICs [8–10]. Ligation of FcγRIIB negatively regulates various immune cells through its inhibitory effects on activating FcγRs, as well as other activating receptors. Ligation of FcγRIIB in conjunction with other receptors, such as the B cell receptor (BCR), inhibits and regulates various immune cells through the tempering of activating signalling. FcγRIIB1 is the main FcγRIIB isoform expressed in B cells. Here, its colocalization with the BCR by antigen:antibody ICs limits B cell expansion and prevents autoimmunity [11, 12], by curtailing BCR signalling, increasing the threshold for B cell activation and reducing B cell-mediated Ag presentation to T cells [13]. In the absence of the BCR, such as on plasma cells, FcγRIIB1 ligation by ICs and subsequent signalling can induce apoptosis, to limit further antibody generation [14]. The ITIM domain (and specifically a single tyrosine residue, phosphorylated following stimulation) in the cytoplasmic domain of FcγRIIB1, is responsible for inhibition of Ca^2+^ flux downstream of BCR stimulation through the activation of phosphatases [2]. Additionally, FcγRIIB1 can inhibit B cell activation by blocking the colocalization of the BCR and CD19 micro-clusters within the immunological synapse, in an ITIM-independent manner requiring the transmembrane domain [15]. Similarly, FcγRIIB1 is critical for limiting self-reactive immune responses during an infection [16]. Therefore, FcγRIIB1 provides three mechanisms pivotal to maintenance of B-cell tolerance: apoptosis of self-reactive cells, follicular exclusion of low-affinity autoreactive B cells and curtailing B cell activation. FcγRIIB2, the main FcγRIIB isoform expressed by myeloid cells, can similarly inhibit their function and negatively regulate responses from activating receptors co-expressed on myeloid cells [2, 17–19] and enforce peripheral tolerance by regulating dendritic cell (DC) function [19]. Amongst others, this has been shown for FcγRIIA (on basophils/monocytes/macrophages) and the IgE receptor FcεRI (on mast cells/basophils) [20]. Moreover, antigen endocytosed by FcγRIIB on DCs accesses a non-degradative intracellular vesicular compartment that recycles to the cell surface, enabling interaction of native antigen with BCR on B cells, thereby enhancing immunization with IgG-opsonized, T-cell-independent antigens [21]. Additionally, high FcγRIIB expression on follicular DCs regulates germinal centre diversity and limits somatic hypermutation by B cells [22]. Therefore, FcγRIIB expression on myeloid cells is of importance for a number of reasons. Firstly, in contrast to the context of the BCR, FcγRIIB2 co-localizes with activating FcγRs including FcγRIIA following binding to ICs, in an antigen non-specific manner. Secondly, the efficacy of monoclonal antibodies (mAb) therapies, which require activating FcγRs for their functions, are limited by myeloid cell FcγRIIB2 (and FcγRIIB1) interactions [3, 23–25].

In preclinical models lacking FcγRII (e.g. FcγRII knockout [KO] mice), immune responses to model antigens and pathogens are significantly elevated [26–32], and FcγRII KO mice are more susceptible to developing autoimmune disorders [33–35]. Similarly, interaction of maternal antibodies with FcγRIIB inhibits B-cell responses during pregnancy [36]; and the levels of FcγRIIB on naïve and marginal zone B cells are reduced in multiple sclerosis patients [37], on leukocytes in patients with systemic lupus erythematosus [38] and on circulatory B cells in Graves’ disease [39]. Importantly, the presence of FcγRIIB can significantly influence anti-tumour cellular and humoral immune responses [40], supports immunoevasion by tumours [41] and hinders the deletion of cancer cells by mAbs [23, 42, 43].

Given these observations, we set out to test whether blocking FcγRIIB with specific mAbs [44–46], would enhance humoral immune responses following immunization with model antigens. We further tested whether these effects were dependent on the engagement of activating FcγRs (by using Fc-null mAbs and FcγR KO mice) or the inhibitory ITIM signalling domain downstream of FcγRIIB (by using NoTIM mice [47]). Our data indicate that treatment of mice with FcγRIIB mAbs prior to immunization significantly boosts humoral immune responses to model antigens as well as potentiating anti-tumour immunity, similar to FcγRII KO mice. Surprisingly, the enhanced immune responses were dependent on the presence and engagement of activating FcγRs by FcγRIIB mAbs, as Fc-null mAbs were inert in the same settings. Furthermore, lack of a functional ITIM in a novel hFcγRIIB Tg mouse model (NoTIM), recently developed by our group [47], failed to affect the responses following FcγRIIB blocking, indicating the redundancy of ITIM-mediated signalling in these experimental settings.

## Materials and methods

### Mice

C57BL/6J and Balb/c mice were purchased from Charles River, UK, and then bred and maintained in local animal facilities, alongside other strains, in accordance with the UK Home Office guidelines. Mouse (m) FcγRII^−/−^, activating mFcγR^−/−^ and human (h) FcγRIIB^−/+^ × mFcγRII^−/−^ mice have been described previously [45, 48]. OT-II TCR Tg mice were sourced from Charles River Laboratories, UK. For NoTIM^+/−^ mice, the ITIM Y273F and Y254F mutations were generated using site-directed mutagenesis from the full length *FCGR2B2* coding region amplified from the human Burkitt’s lymphoma Raji cell cDNA and introduced into the mouse genome through microinjection of C57BL/6J oocytes by Cyagen, as previously described [47]. NoTIM^+/−^ and hFcγRIIB^+/−^ mice were inter-crossed with mFcγRII^−/−^ mice (C57BL/6J) to remove the endogenous mouse inhibitory receptor. NoTIM progenies were screened by PCR (amplifying genomic DNA extracted from ear tips) or flow cytometry of peripheral blood. All experiments were conducted under UK Home Office licenses PPL30/1269 and P4D9C89EA and following approval by local ethical committees, reporting to the Home Office Animal Welfare Ethical Review Board (AWERB) at the University of Southampton. Experiments used both male and female mice which were age- and sex-matched within experiments. For the majority of experiments mice were aged between 8-16 weeks. Littermates of the same sex were randomly assigned to experimental groups at the start of the experiment. Food (irradiated RM1 (E)) and water was available ad libitum, mice were maintained on a 12-hour light/dark cycle and environmental enrichment was provided; temperature was maintained between 20 and 24^o^C.

### Immunization protocols

Age- and sex-matched WT mice were intraperitoneally (I.P.) injected with 0.5 mg isotype control (iso ctrl; mIgG1) or mouse FcγRII mAbs (clones AT128 and AT130-5 [mouse IgG1] [44, 49]) or human FcγRIIB mAb (clone BI-1206 [human IgG1] [45]) on day -1, followed by intravenous (I.V.) injection of 0.5 mg chicken ovalbumin (OVA; Sigma, UK), spiked with 2-10 µg LPS (Sigma, UK), on day 0. Mice were bled 7, 14 and 28 days post immunization and serum anti-OVA IgG levels were measured by ELISA, as before [50]. Mice were then rechallenged with 50 µg OVA (I.V.) and anti-OVA IgG assayed over the subsequent 28 days as above. In experiments where OT-II CD4^+^ T cells were assayed, a total of 2 × 10^6^ splenocytes from an OT-II mouse was injected (I.V.) 1 day prior to immunization. For CD4^+^ T cell depletion experiments, mice were injected with a cocktail of CD4-depleting mAbs (500 µg; clones GK1.5 and YTA3.1.2) on Day −7 and Day −3, prior to immunization, as before [51]. For macrophage depletion experiments, clodronate liposomes were generated as before [52]. Freshly prepared clodronate liposomes or PBS liposomes were then injected (I.V.) into mice on Days −3 and −1, before the initiation of immunization.

### Cell lines

B16-OVA melanoma cells were maintained in Dulbecco’s modified Eagles Medium supplemented with glutamine (2 mM), pyruvate (1 mM), penicillin and streptomycin (100 IU/mL), amphotericin (2 mg/mL) and 20% foetal calf serum (FCS), and maintained as before [51].

### Antibodies

Anti-human FcγRIIB hIgG1 mAb (clone 6G11 [BI-1206]) and its N297Q mutant (clone BI-1607), were generated by BioInvent International AB, as previously described [45, 47]. Anti-mouse FcγRII mIgG1 mAbs (clones AT128 and AT130-5) were produced in-house using stably transfected CHO-K1 cells, as previously described [44, 49]. Purity was assessed by CE-SDS electrophoresis (Beckman EP; Beckman, USA) and lack of aggregation confirmed by size exclusion high performance liquid chromatography. Unless otherwise stated, all antibodies were administered I.V. or I.P. in 200 µL sterile PBS.

Flow cytometry antibodies used were as follows: B220-PerCP (clone RA3-6B2), CD19-APC (clone HIB19) and CD138-PE (clone 281-2) were all sourced from BioLegend, UK. For OT-II cell staining, anti-mouse Vα2–TcR-FITC (clone B20.1), anti-mouse Vβ5.1,5.2 TcR-PE (clone MR9-4), and allophycocyanin-labelled anti-CD4 (clone RM4-5; all from BD Biosciences, USA) were used, as before [50]. Allophycocyanin-labelled F4/80 antibody (rat IgG2b; clone CI:A3-1) was purchased from BioRad, UK.

### Flow cytometry

Samples were stained with the appropriate antibody-fluorophore conjugate for 30 minutes at 4^o^C in the dark. Samples were then washed in Erytholyse red blood cell lysing buffer (BioRad, UK) and subsequently washed in FACS buffer (PBS, 1% BSA, 0.1% sodium azide). Samples were stored in the dark at 4^o^C until analysis. FACSCalibur and FACSCanto II flow cytometers were used for data acquisition (BD Biosciences, USA).

### Immunofluorescence

Tissues were frozen in OCT media (Cellpath, UK) and placed in isopentane on a bed of dry ice. 10 μm frozen sections were then cut, fixed in acetone and blocked with 5% normal goat serum before incubation with F4/80 antibody followed by goat anti-rat-AF488 (Invitrogen, UK). Slides were mounted using Vectashield hardset with 4′,6-diamidino-2-phenylindole (DAPI; Vector Laboratories, UK). Images were collected with a CKX41 inverted microscope using a Plan Achromat 10 × 0.25 objective lens (Olympus, Japan). RGB images (TIFF) were transferred to Adobe Photoshop CS6 and RGB image overlays created. Background autofluorescence was removed, contrast stretched, and brightness adjusted to maximize clarity, with all images treated equivalently.

### Quantification and statistical analysis

Flow cytometry data analysis was performed using FlowJo version 10.6 (BD Biosciences, USA). All other data analysis was performed using GraphPad Prism versions 7-9 (GraphPad Software, USA). Statistical significance between two factors was analysed using a two-tailed unpaired *t*-test. The statistical significance in long term survival experiments was analysed using Kaplan–Meier survival test with the Mantel–Cox test used to assess significance between groups. Throughout, * *P* < 0.05, ** *P* < 0.01, *** *P* < 0.001.

## Results

### Mouse FcγRII mAbs enhance immune responses in WT mice

**Figure 1:**
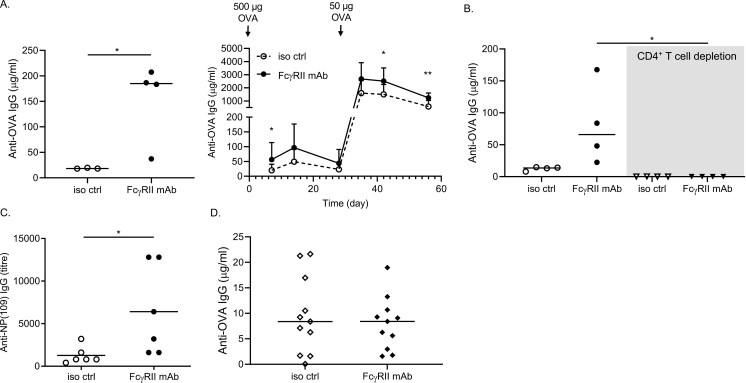
FcγRII mAbs enhance humoral immune responses in WT mice and depend on activating FcγRs. **(A)** Age- and sex-matched WT mice were injected with 0.5 mg isotype control (iso ctrl; mIgG1) or FcγRII mAb (I.P.) on Day −1, followed by 0.5 mg chicken OVA + LPS (I.V.) on day 0. Serum anti-OVA IgG levels were measured by ELISA on days 7, 14, and 28. Mice were then rechallenged with 50 µg OVA and anti-OVA IgG assayed, as before. Representative data for Day 7 post primary immunization shown on the left and longitudinal data shown in the right panel. Mean + SD shown (*n* = 3 independent experiments; 8–16 mice/group). **(B)** CD4^+^ T cells were depleted prior to the immunization of mice and serum anti-OVA IgG was assessed as above (Day 14 results shown). **(C)** Age- and sex-matched WT mice were injected with 0.5 mg isotype control (iso ctrl; mIgG1) or FcγRII mAb (I.P.) on Day -1, followed by NP(109) on Day 0. Serum anti-NP(109) IgG was assessed post immunization (Day 14 results shown). **(D)** Age- and sex-matched mice lacking activating FcγRs (FcγR^−/−^) were injected with 0.5 mg isotype control (iso ctrl; mIgG1) or FcγRII mAb (I.P.) on Day −1, followed by 0.5 mg chicken OVA (I.V.) + LPS on Day 0. Serum anti-OVA IgG levels were measured by ELISA, as before (Day 7 results shown). Each dot represents an individual mouse; * *P* < 0.05, ** *P* < 0.005

In order to investigate whether targeting FcγRII was capable of enhancing immune responses, WT mice were treated with an irrelevant isotype control mAb or anti-mouse FcγRII mAbs prior to immunization with the model antigen OVA. FcγRII mAb pre-treatment led to a significant increase in serum anti-OVA IgG following both primary and secondary immunization (Fig. 1A). OVA is a T-cell-dependent antigen and, in agreement with this, depleting CD4^+^ T cells prior to OVA immunization abrogated immune responses to OVA, regardless of FcγRII mAb treatment (Fig. 1B). Similarly, depletion of macrophages by clodronate liposomes (Supplementary Fig. 1A) prior to immunization led to a ~2-fold reduction in anti-OVA serum IgG levels (Supplementary Fig. 1B). Despite this, blocking FcγRII mAb was still capable of boosting anti-OVA IgG responses post immunization, albeit at lower levels in mice lacking macrophages (Supplementary Fig. 1). This is likely due to the blocking of FcγRII on B cells, that lack activating FcγRs, thereby potentiating BCR responses.

We further tested whether mouse FcγRII mAbs were capable of potentiating immune responses to a T cell-independent antigen, NP-AECM-FICOLL (referred to as NP(109)). As with OVA immunization, pre-treatment of mice with FcγRII mAbs significantly enhanced serum—antibodies against NP(109), suggesting that blocking FcγRII boosts humoral responses to both T cell-dependent and -independent antigens *in vivo* (Fig. 1C).

### FcγRII mAbs fail to potentiate immune responses in the absence of activating FcγRs

Antibodies are composed of two key components: The F(ab) which recognizes the target antigen and Fc, which engages FcγRs on leukocytes, such as macrophage and natural killer cells [53]. As such, WT FcγRII mAbs can engage activating FcγRs via the Fc after binding FcγRII through the F(ab) regions. To investigate whether activating mouse FcγRs (namely FcγRI, FcγRIII and FcγRIV) were involved in the FcγRII mAb-mediated enhancement of humoral immune responses after immunization, activating FcγR KO mice were immunized as above. To our surprise, the absence of activating FcγRs abrogated the enhancement of humoral responses by FcγRII mAb (Fig. 1D). This suggests that once bound to FcγRII by the F(ab), the Fc interacts with activating FcγRs on effector cells to elicit stronger humoral responses.

### FcγRII mAbs enhance anti-tumour immune responses

**Figure 2:**
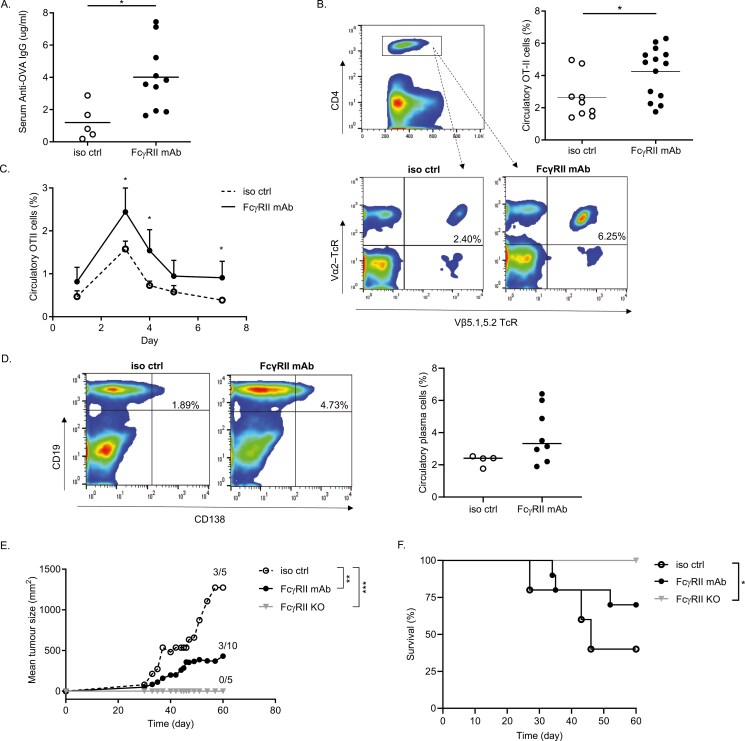
FcγRII blockade potentiates expansion of antigen-specific CD4^+^ T cells and reduces tumour growth in OVA-immunized mice. Age- and sex-matched WT mice were injected with OT-II splenocytes (2 × 10^6^ cells; I.V.) and 0.5 mg isotype control (iso ctrl; mIgG1) or FcγRII mAb (I.P.) on Day −1, followed by 0.5 mg chicken OVA + LPS (I.V.) on Day 0. Serum anti-OVA IgG levels were measured by ELISA on Days 7, 14, and 28, as before. **(A)** FcγRII mAb treatment resulted in a significant increase in serum anti-OVA IgG levels (Day 14 results shown), **(B–C)** circulatory antigen-specific T cells (OT-II cells) and **(D)** a considerable expansion of CD19^+^CD138^+^ plasma cells (Day 4 post primary immunization). Day 3 results of a representative experiment shown in **(B)**. **(E–F)** B16-OVA melanoma cells (5 × 10^5^ cells) were subcutaneously engrafted into immunized mice and tumour growth was monitored over time **(E)**. **(E)** Mean (+ SD) of a representative experiment shown (*n* = 2); tumour volumes were compared on day 60 using a two-tailed unpaired *t*-test. **(F)** Mice from **(E)** were monitored over time and sacrificed upon evidence of terminal tumour development. Survival was compared using the Mantel–Cox test; * *P* < 0.05. **(A–D)** Each dot represents an individual mouse; * *P* < 0.05. **(B and D)** Representative flow cytometry plots for circulatory OT-II CD4^+^ T cells and plasma cells shown, respectively. **(A-F)** * *P* < 0.05, ** *P* < 0.005, *** *P* < 0.001

We next explored whether FcγRII mAbs could enhance cell-mediated and/or anti-tumour immune responses. To assess the cellular response, we injected WT mice with OT-II Tg CD4^+^ T cells that specifically recognize OVA peptides, and then immunized them with OVA in the presence or absence of FcγRII mAb. As hypothesized, FcγRII mAb treatment significantly enhanced humoral immune responses (Fig. 2A) and antigen-specific CD4^+^ T cells (Fig. 2B-C) *in vivo*. Furthermore, in-line with the increase in anti-OVA IgG levels, FcγRII mAb-treated mice had an expansion in CD19^+^CD138^+^ plasma cells post immunization (Fig. 2D), albeit not reaching statistical significance.

To evaluate anti-tumour immune responses, WT and FcγRII KO mice were immunized as before, and then subcutaneously injected with B16-OVA melanoma cells and then monitored over time. Tumour growth was significantly retarded in FcγRII KO mice and WT mice treated with FcγRII mAbs, compared with mice that received an irrelevant isotype control mAb (Fig. 2E). This translated into a significant increase in the survival of FcγRII KO mice and a modest increase in the survival of FcγRII mAb-treated WT mice, compared with isotype control-treated WT mice (Fig. 2F). Collectively, these results indicate that deletion, or blockade of FcγRII can potentiate anti-tumour immune responses.

### WT but not Fc-null FcγRIIB mAbs enhance humoral immune responses

**Figure 3:**
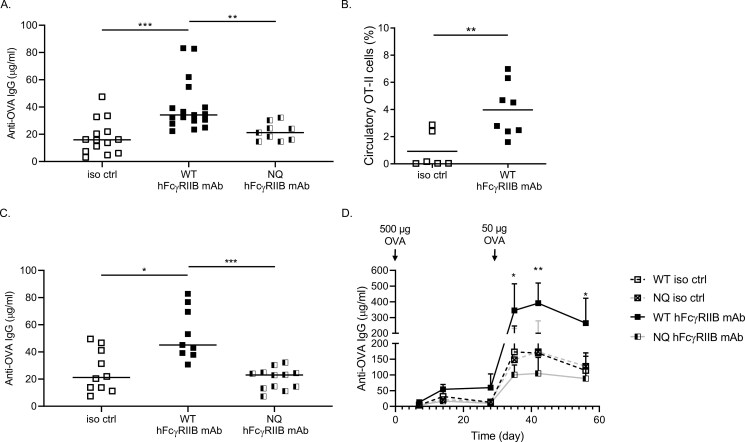
WT but not Fc-null FcγRIIB mAbs enhance immune responses in hFcγRIIB Tg mice, independent of ITIM signalling. **(A)** Age- and sex-matched hFcγRIIB Tg mice were injected with 0.5 mg isotype control (iso ctrl; mIgG1) or WT or Fc-null (NQ) human (h) FcγRIIB mAb (I.P.) on Day −1, followed by 0.5 mg chicken OVA + LPS (I.V.) immunization on Day 0. Serum anti-OVA IgG levels were measured by ELISA on Days 7, 14, and 28. Mice were then rechallenged with 50 µg OVA and anti-OVA IgG was assayed, as before. Human FcγRIIB mAb significantly enhanced immune responses compared with the isotype control-treated group (Day 14 results shown). Each dot represents an individual mouse (*n* = 3 independent experiments). **(B)** Circulatory OT-II CD4^+^ T cells were assayed by flow cytometry, as above (Day 3 post-primary immunization shown). **(C–D)** Age- and sex-matched NoTIM mice were injected with 0.5 mg isotype control (iso ctrl; mIgG1) or WT or Fc-null (NQ) human FcγRIIB mAb (I.P.) on Day −1, followed by 0.5 mg chicken OVA + LPS (I.V.) immunization on Day 0. Serum anti-OVA IgG levels were measured by ELISA on Days 7, 14, and 28. Mice were then rechallenged with 50 µg OVA and anti-OVA IgG was assayed, as before. Representative data for Day 14 post-primary immunization shown in **(C)** and longitudinal data shown in **(D)**. Mean + SD shown. Each dot represents an individual mouse; * *P* < 0.05, ** *P* < 0.005, *** *P* < 0.001.

Next, we sought to test the ability of human FcγRIIB mAbs to enhance antibody responses in a hFcγRIIB Tg mouse model [45]. Here, we tested both the WT mAb and Fc-null variant of the same clone (6G11) containing the N297Q mutation [54]. The latter would be expected to engage FcγRIIB but not activating FcγRs, in a model akin to the activating FcγR KO mice, described above. As above, treatment of hFcγRIIB Tg mice with the WT mAb prior to immunization led to a significant enhancement of anti-OVA IgG levels; whereas, the Fc-null mAb failed to cause any increase (Figure 3A). Similarly, the WT human FcγRIIB mAb treatment enhanced OT-II CD4^+^ T cell expansion *in vivo* (Figure 3B), indicating the potential of FcγRIIB mAb to enhance immune responses and vaccination-induced antibody titres *in vivo*.

### FcγRIIB mAb enhancement of immune responses is not dependent on the FcγRIIB ITIM

**Figure 4.**
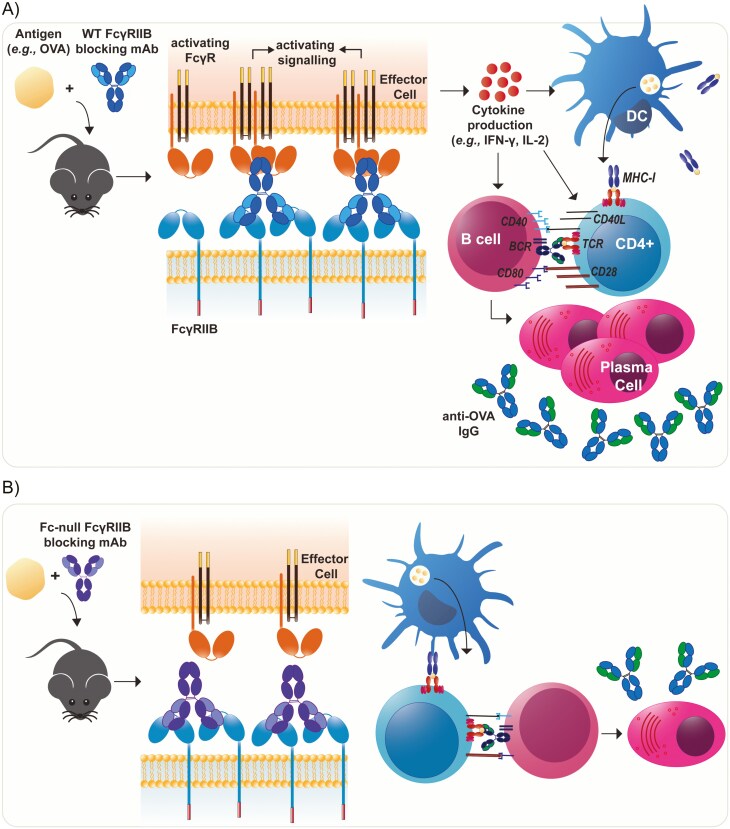
Schematic diagram demonstrating the proposed mode of action of FcγRIIB mAbs *iv vivo*. **(A)** Blocking FcγRIIB with WT FcγRIIB mAbs results in crosslinking of neighbouring activating FcγRs and enhancement of immune responses following immunization. **(B)** Fc-null FcγRIIB mAbs, which fail to engage activating FcγRs on neighbouring effector cells, e.g. macrophages and DCs, fail to enhance immune responses to antigens.

Given the opposing effects of the two FcγRIIB mAb formats and dependence of the augmentation effect on activating FcγR expression/interaction, we next addressed whether this activity was dependent on the ITIM signalling capacity of FcγRIIB, using hFcγRIIB Tg mice containing a defective ITIM (NoTIM). NoTIM mice were treated with either WT or Fc-null FcγRIIB mAbs and immunized with OVA, and then their primary and secondary humoral response evaluated. Similar to the responses observed in hFcγRIIB Tg mice (Fig. 2), WT but not Fc-null human FcγRIIB mAb enhanced both primary and secondary anti-OVA IgG responses in NoTIM mice (Fig. 3C–D). These data strongly suggest that the enhancement of immune responses is independent of inhibitory ITIM signalling, and instead are Fc dependent. Once the F(ab) is bound to FcγRIIB, the Fc engages activating FcγRs to augment the vaccinal response (Figure 4).

## Discussion

The immune response to infection is tightly regulated to ensure optimal defence from pathogens whilst avoiding the consequences of excessive inflammation. Likewise, negative immunoregulatory circuits comprised of inhibitory molecules exist to prevent the onset of autoimmune disorders by suppressing autoreactive lymphocytes. FcγRIIB is one such immunoregulatory molecule, whose expression on both myeloid and lymphoid cells modulates humoral and cellular immune responses [2, 55]. Deleting, down-regulating or blocking of FcγRIIB modifies immune responses during both steady state and infection, with FcγRIIB deficiency contributing to a number of pathologies [14, 15, 18, 26–32, 35, 37, 40]. Expectedly, FcγRII KO mice exhibit elevated immune responses and are prone to the development of a range of autoimmune disorders [12, 26, 33, 34], which is believed to be due to hyperactivation of B cells and myeloid cells.

Since genetic targeting of FcγRIIB *in situ* is not yet practical, using previously generated specific mAbs against mouse FcγRII [44, 49] and human FcγRIIB [45, 47], we set out to examine whether mAb targeting of FcγRIIB would enhance humoral immune responses in relevant preclinical models. WT mAbs have a Fc domain, which facilitates engagement with FcγRs on leukocytes, in particular myeloid effector cells. Hence, we sought to compare WT and Fc-null (deglycosylated Fc due to introduction of a N297Q point mutation) mAbs [54] for their ability to enhance immune responses. To our surprise, the Fc-null mAb failed to augment vaccine responses, suggesting that blocking the activity of FcγRIIB was less important than engagement of activating FcγRs by the mAb Fc. This was further confirmed by immunizing FcγR KO mice, which lack all activating FcγRs but retain FcγRII [52]. Unlike in WT mice, targeting of FcγRII in activating FcγR KO mice failed to modulate humoral immune responses against the experimental antigen. Collectively, these complementary models indicate that engagement of activating FcγRs is central to the effects seen above and suggest that the blocking of the inhibitory FcγRII by mAb per se was not responsible for the enhanced vaccinal effects seen.

FcγRIIB signals through its intracellular ITIM domain, to deliver its inhibitory signals and impede immune responses [2, 7]. To test whether blocking of FcγRIIB-mediated inhibitory signalling using specific mAbs contributes to the augmented vaccinal response, we utilized the NoTIM mouse model [47]. As before, WT FcγRIIB mAb induced a significant increase in humoral responses post primary and secondary immunizations in NoTIM mice, whereas the Fc-null mAb was inert. These findings are contrary to our initial hypothesis, where we expected that FcγRIIB blocking mAbs (WT and Fc-null) would limit FcγRIIB engagement of ICs and thereby potentiate BCR and FcγR-mediated responses in B cells and myeloid cells (e.g. DCs and macrophages), respectively. Although deficiency in FcγRIIB potentiates immune responses via the reduction in inhibitory signals, FcγRIIB mAbs were shown to confer a similar effect via a totally different mechanism, namely by engaging activating FcγRs via their Fc domain. This phenomenon has been shown in other experimental systems and is referred to as the ‘scorpion’ or ‘Kurlander’ effect, where the antibody Fc is able to bind to proximal FcγRs [56–59]. Indeed, this effect is commonly observed on cells expressing FcγRs, responsible for inhibiting (e.g. anti-CD20) or potentiating (e.g. anti-CD40) activity through stabilization of the mAb via Fc binding to proximal FcγRIIB [25, 43, 47, 50, 60–64]. Therefore, these data have important implications for engineering optimal therapeutic mAbs. For immunostimulatory mAbs, a WT format may be optimal in order to enhance their crosslinking capacity. On the other hand, where blocking of the target is desired, Fc-null mAbs may be superior. In the case of FcγRIIB and boosting responses following immunization, our data suggest that WT mAb format would be the preferred choice, given that Fc-null mAb was inert.

Our study has a number of limitations, including the choice of immunogen, route of immunization and limited number of FcγRIIB mAbs assessed. We mainly utilized chicken OVA, found in egg whites, as the main model antigen, due to the availability of the reagents and preclinical models. However, while OVA can be used to study basic principles of the immune system, it may not fully recapitulate immune responses to more physiological antigens. The applicability of our findings needs to be determined through further testing of FcγRIIB mAbs with these antigens, in models of infection. Moreover, the intravenous immunization route employed in this study differs from typical human vaccination methods, such as intramuscular or intranasal administration. Finally, our investigation focused solely on the antagonistic human FcγRIIB antibody clone 6G11 (BI-1206) [45]. To gain a more comprehensive understanding of optimal FcγRIIB targeting for immunization, future studies should incorporate additional clones recognising distinct epitopes with varying functional effects, including agonistic clones [45]. Although we observed similar responses with both antagonistic (AT128) and agonistic (AT130-5) mouse FcγRII antibodies [44] (data not shown), further investigation with a broader range of human clones is warranted.

## Conclusion

In summary, our data show that WT anti-mouse FcγRII and anti-human FcγRIIB mAbs can augment humoral immune responses via interaction of their Fc with activating FcγRs. They further reveal that the enhancement of immune responses is not a direct consequence of impeding FcγRIIB ITIM-mediated signalling. Rather the F(ab) binding to FcγRIIB acts as an anchor to promote the engagement of neighbouring (*cis* or *trans*) activating FcγRs via their Fc domain, in order to initiate a more potent immune response. Although FcγRIIB is expressed by both B cells and myeloid cells, since B cells lack activating FcγRs, it is expected that the enhancement of immune responses is primarily a consequence of engagement of activating FcγRs on myeloid cells. This was evident when macrophages were depleted in a cohort of immunized mice, where there was a ~50% reduction in anti-OVA antibodies. Regardless of the mode of action of the FcγRIIB antibodies, our study provides a strong basis for the therapeutic application of FcγRIIB mAbs to promote immune responses against antigens, such as tumour-associated and non-self antigens. A human FcγRIIB mAb, previously developed by us [45], is currently in Phase I/IIa clinical trials (Clinical trial #NCT03571568 and NCT04219254). This antibody, which has been granted Orphan Drug Designation by the FDA for the treatment of follicular lymphoma and exhibited clinical benefit in other refractory lymphoma patients [65], has the potential for broader clinical applications, including vaccine enhancement.

## Supplementary Material

uxaf015_suppl_Supplementary_Figure_S1

## Data Availability

All data are available within the manuscript and any additional data are available upon request.
