# Supplementary figures and images for: FcγRIIB (CD32B) antibodies enhance immune responses through activating FcγRs
Source: Clin Exp Immunol. 2025 Mar 16;219(1):uxaf015. doi: 10.1093/cei/uxaf015 (PMC12046127; doi:10.1093/cei/uxaf015)

Supplementary Figure 1.

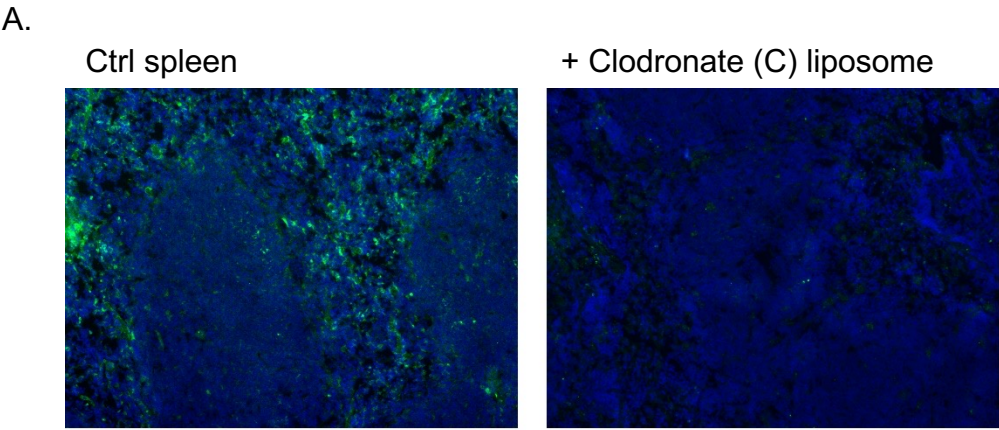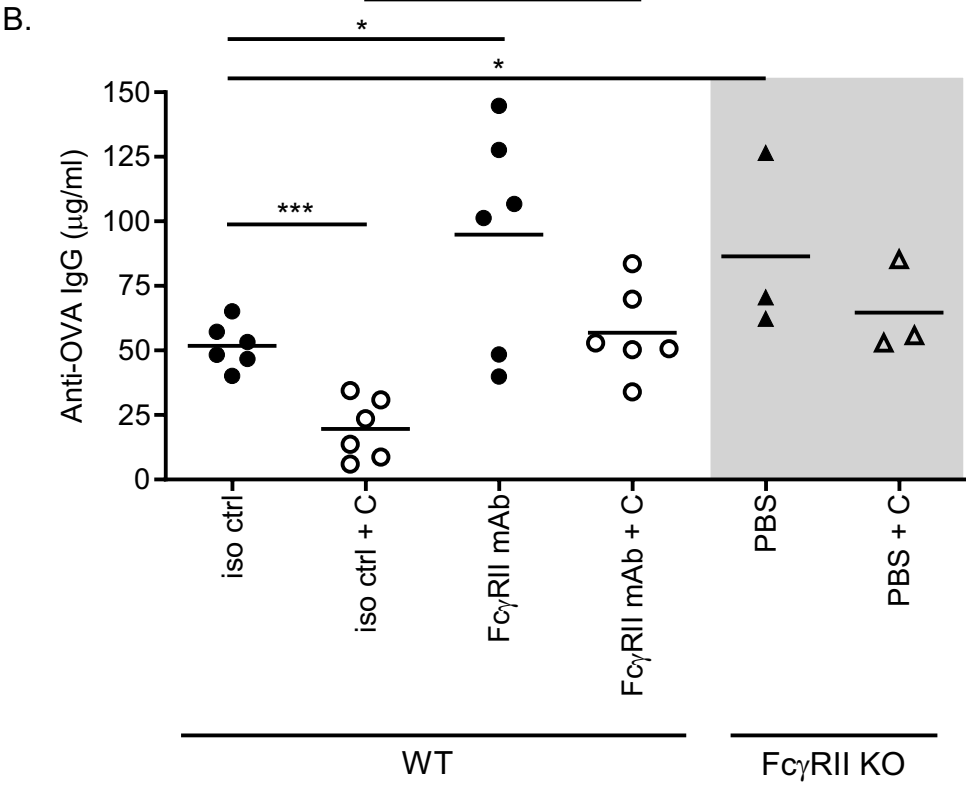

Supplement: uxaf015_suppl_Supplementary_Figure_S1 [file uxaf015_suppl_supplementary_figure_s1.pdf]
